# Coping With Diabetes During the COVID-19 Lockdown in Saudi Arabia: Lessons Learned in the Post-pandemic Era

**DOI:** 10.7759/cureus.31522

**Published:** 2022-11-15

**Authors:** Adnan Alharbi, Areej Alduribi, Ahad Alghthami, Mohamed Elnaem, Faisal S Alsenani, Abdul Haseeb, Nehad J Ahmed, Mahmoud Elrggal

**Affiliations:** 1 Clinical Pharmacy, Umm Al-Qura University, Makkah, SAU; 2 Pharmacy Practice, International Islamic University Malaysia, Kuantan, MYS; 3 Pharmacy, Umm Al-Qura University, Makkah, SAU; 4 Clinical Pharmacy, Prince Sattam Bin Abdulaziz University, Dammam, SAU

**Keywords:** self-monitoring, pandemic, healthcare services, diabetes mellitus, covid-19

## Abstract

Background: Uncontrolled diabetes has appeared as one of the major risk factors for morbidity and mortality in diabetic patients with coronavirus disease 2019 (COVID-19). Alterations in dietary habits, physical inactivity, and inability to take advice from the physician are some of the contributing factors. This study aimed to assess the impact of the COVID-19 lockdown in Saudi Arabia on medication accessibility, medication adherence, lifestyle, and quality of life of diabetes patients.

Methods: A cross-sectional observational study was conducted among diabetic patients using a self-reported questionnaire developed on an online platform (SurveyMonkey®). The survey was distributed through social media platforms (WhatsApp, Telegram). For those who were digitally illiterate, responses were collected by family members. The targeted population was type 1, type 2 and gestational diabetes patients. The analysis of the data was done using IBM SPSS Statistics, version 26.

Results: Four hundred forty-nine participants completed the survey. Most of the participants had type 2 diabetes (n=359; 79.8%) and were well educated (83.2%) with a high school degree and above. Complications from COVID-19 infection were reported in 12% (n=54) patients. During quarantine, 78.8% (n=354) of participants measured their blood glucose regularly. Results showed that during quarantine, 68.3% (n=311) participants skipped their scheduled follow-up whereas only 5.1% (n=23) of them took their medication inappropriately.

Conclusion: This study reported good levels of self-monitoring of blood glucose levels, whereas patients’ accessibility to seek healthcare services seemed to be interrupted. Further efforts are needed in the post-pandemic era to empower patients’ self-care behaviors and utilize telehealth models to facilitate timely access to medical care.

## Introduction

In December 2019, severe cases of pneumonia were reported due to a new type of virus, the novel coronavirus leading to coronavirus disease 2019 (COVID-19). COVID-19 rapidly spread all over the world. On March 11, 2020, the World Health Organization declared COVID-19 as a global epidemic [1]. As a worldwide response, the governments took actions to stop the spread of COVID-19 by closing schools and workplaces and canceling significant public events [2]. In addition to all those actions, they restricted public transport and movement of people and ceased domestic and international flights [3]. On March 2, 2020, the first COVID-19 case in Saudi Arabia was announced, which led to an immediate action from Saudi Arabia's government that started on March 4, imposing a nationwide partial lockdown (from 7 PM to 6 AM), with movement between provinces restricted and suspended international flights; finally, a complete lockdown was imposed from April 6 to May 28 [4].

According to the World Health Organization, diabetes will be the seventh leading cause of death by 2030 [5]. The 10th edition of the International Diabetes Federation Atlas documents an ongoing rise in diabetes prevalence worldwide, indicating that the disease poses a serious threat to the health and well-being of individuals, families, and societies. The report showed that in the Middle East and North Africa, 73 million adults have diabetes. The report also showed that one-third of persons with diabetes who are living with it are undiagnosed [6]. Diabetes prevalence in Saudi Arabia is high according to data published in many studies. Alqurashi et al. stated that the prevalence of diabetes was 34.1% in males and 27.6% in females [7]. In addition, other study results showed that around 50% of males and females aged 50 years had diabetes [8]. Moreover, Saudi Arabia and other Middle Eastern countries have a high prevalence of obesity that affects the prevalence of diabetes mellitus negatively [9]. The anti-diabetic medications seem hard to adhere to because of their tough dosage regimen plan and polypharmacy. However, several published studies explored the public knowledge and awareness related to adherence to diabetic medications. A study done in Saudi Arabia showed that the percentage of patients who were not taking their medications as prescribed was around 56%. This non-compliance and poor adherence affect the optimal glycemic status of patients that ultimately increases the diabetic complications [10]. Coronary heart disease, stroke, nephropathy, and retinopathy affect 25% of patients with type 2 diabetes mellitus. Neuropathy seems to affect almost 50% of patients and erectile dysfunction affects 35%-90% of diabetic men [11].

People with diabetes are more vulnerable to COVID-19 development. The risk of complications and mortality is even higher among older people with diabetes [12]. There is an evidence that the incidence and severity of COVID-19 increased in patients with diabetes because the novel coronavirus shared common pathophysiology with diabetes [13]. Landstra and de Koning found that a severe COVID-19 infection, and its treatment with steroids, can have a specific negative impact on diabetes itself, leading to the worsening of hyperglycemia through increased insulin resistance and reduced β-cell secretory function [14]. Apicella et al. found that individuals with type 1 diabetes are also at risk of severe COVID-19. And that severe acute respiratory syndrome coronavirus 2 (SARS-CoV-2) infection itself might represent a worsening factor for people with diabetes, as it can precipitate acute metabolic complications through direct negative effects on the β-cell function [15]. Nevertheless, there is limited data about the impact of COVID-19 on diabetes and medication compliance during the lockdown. This study aimed to describe the medication accessibility, medication adherence, lifestyle, and quality of life of diabetes patients under the COVID-19 lockdown in Saudi Arabia.

## Materials and methods

Study design

A cross-sectional observational study was conducted among diabetic patients. A self-reported questionnaire was made of 42 questions after a thorough literature review. The questionnaire was developed on an online platform (SurveyMonkey®; Momentive Inc., San Mateo, CA). It was distributed through social media platforms (WhatsApp, Telegram, etc.). For those who were digitally illiterate, responses were collected by family members.

The study included type 1, type 2 and gestational diabetes patients in Saudi Arabia. So, patients who didn’t have diabetes and patients who had prediabetic glucose level were excluded from the study.

Instrument of measure

The survey consisted of four sections: demographic information, knowledge of diabetic patients about their disease, attitude, and awareness of diabetic patients toward their disease and diabetic care during COVID-19 lockdown. The survey was validated by experts from academics and healthcare providers. The experts ensured that the survey instrument's data was reliable and that the survey had face and content validity. The demographic information section data included questions on age, gender, height and weight, occupation, marital status, and level of education. In the section about disease knowledge of diabetic patients, patients were asked about the type of diabetes (type 1, type 2, gestational diabetes), if they had any comorbid conditions (dyslipidemia, heart disease, eye disorder, kidney disease, circulation disease), the duration of the disease, medication type (insulin, tablet, both) and other questions to assess the knowledge about their disease. The third section composed of 11 questions in order to evaluate the attitude and awareness of diabetic patients toward their disease, including the clinical symptoms of low blood glucose, times of measuring blood glucose, hemoglobin A1c (HbA1c) level, and the importance of taking the medication as prescribed. The last section composed of 12 questions to describe diabetic care during the COVID-19 lockdown in Saudi Arabia. All questions were retrospective in nature and assessed patients' perspective on the change happened in their disease status.

Sample size calculation

We used Raosoft calculator (Raosoft, Inc., Seattle, WA) to determine the recommended sample size. The minimum recommended size of the survey was 385 (using 5% margin of error, confidence level of 95%, and response distribution of 50%).

Data collection

The respondents were asked if they got COVID-19 or if a family member had been infected with COVID-19. Respondents were asked additional questions about their adherence to medications and monitoring blood glucose, physical exercise, and if they worked outside the house during the lockdown. Furthermore, the questions related to the challenges and the barriers that might have prevented them from taking the medication during quarantine, as prescribed, were also asked. Participants were asked to respond in a seven-point Likert scale response option ranging from 1 (strongly agree) to 7 (strongly disagree) and reflect on their feelings and thoughts for each item on the scale, during quarantine.

Statistical analysis

The analysis of the data was done using IBM SPSS Statistics, version 26 (IBM Corp., Armonk, NY). A descriptive analysis was conducted, and the data were presented as frequencies and percentages for the responses. The analysis of variance (ANOVA) tests were used to find the association between two different variables. The probability value (p-value) was less than 0.05 and was statically significant.

Ethical consideration

The study was conducted in accordance with the Declaration of Helsinki, and approved by the Institutional Review Board of Umm Al-Qura University, Makkah, Saudi Arabia (IRB number HAP-02-T-067). Researchers explained the nature of the study to the potential participants in the survey. Data was totally anonymous and names or any personal information was not disclosed.

## Results

Demographics

**Figure 1:**
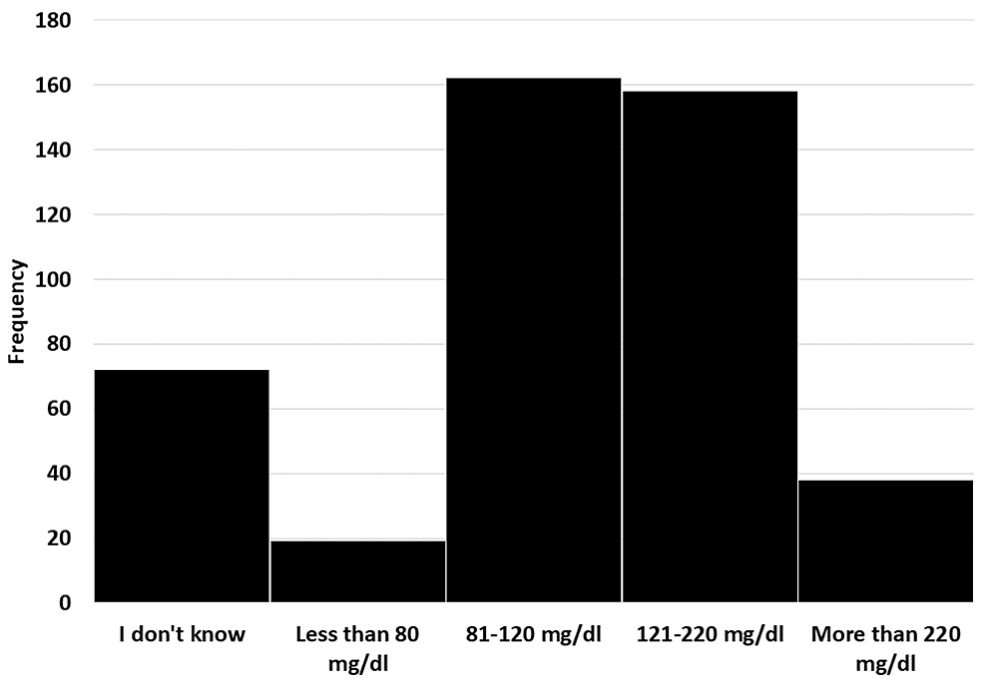
Response to survey question, 'How high is your fasting blood glucose measurement?'

**Figure 2:**
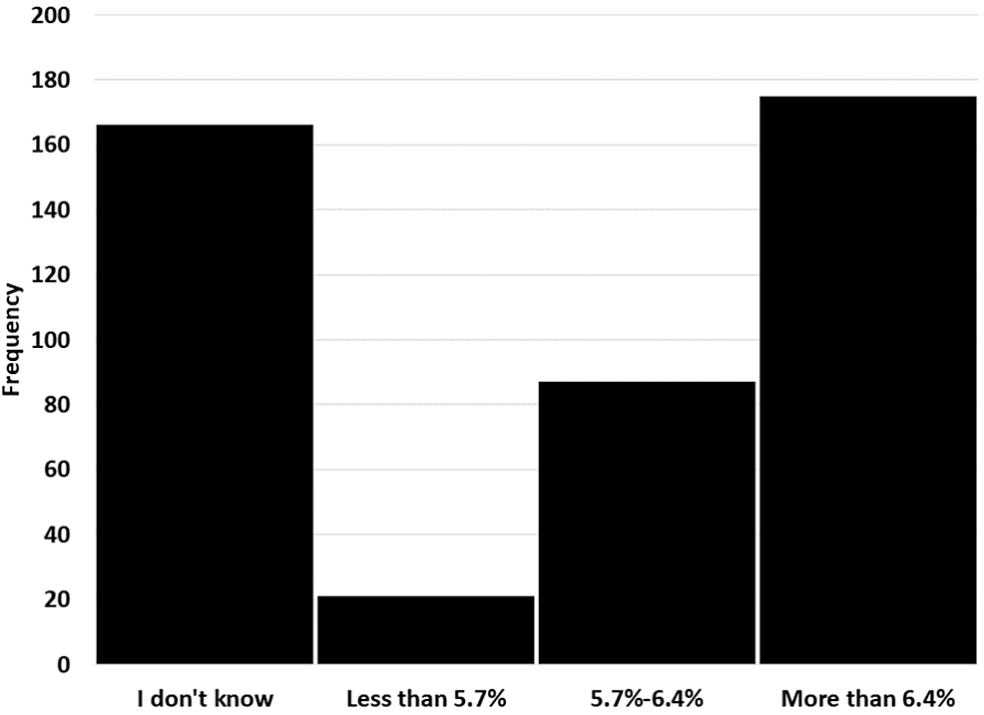
Response to survey question, 'How high is your HbA1c?'

The survey was sent to diabetic patients only. All responses were counted and included in the results. Four hundred forty-nine participants completed the survey. The socio-demographic characteristics of 449 diabetes patients were included in the final analysis with a mean age of 42.45±15.40 years, ranging from 8 to 92 years. The average body weight was 76.41±19.84 kg, while the height was 160±10.78 cm. Most of the participants were female (64.1%) and married (68.6%). It also showed that a high number of the participants were well educated (83.2%) with a high school degree and above. The data showed that 31.6% were employees, 17.6% were students, and 17.6% were retired. Most of the participants had type 2 diabetes (79.8%). The duration of diabetes was more than 10 years in 178 (39.6%) patients. Around 55% patients were taking pills while 28.2% were on insulin therapy (Table 1, Figure 1). Almost half of the participants 178 (39.6%) had their HbA1c level above 6.4% (Figure 2).

**Table 1:** Demographic information BSL: blood sugar level

Parameters	Findings
Gender	
Male	161 (35.9%)
Female	288 (64.1%)
Age	42.45±15.40
Weight	76.41±19.84
Height	160±10.78
Marital status	
Single	13 (13.4%)
Married	78 (80.4%)
Divorced	3 (3.1%)
Widow	3 (3.1%)
Occupation	
Unemployment	38 (39.2%)
Employee	27 (27.8%)
Student	11 (11.3%)
Retired	21 (21.6%)
Comorbid conditions	
Dyslipidemia	36 (37.1%)
Heart disease	13 (13.4%)
Eye disorder	44 (45.4%)
Kidney disease	7 (7.2%)
Circulation disease	3 (3.1%)
No comorbidity	21 (21.6%)
Others	9 (9.3%)
Type of diabetes	
Type 1	67 (14.92%)
Type 2	359 (79.96%)
Gestational	23 (5.12%)
Duration	
1-5 years	124 (27.62%)
5-10 years	147 (32.74%)
>10 years	178 (39.64%)
Type of medications that patients took	
Insulin injection	127 (28.29%)
Oral medication	247 (55.01%)
Both	75 (16.70%)
BSL level	
<80	18 (4%)
81-120	162 (36.04%)
121-220	158 (35.19%)
>220	38 (8.46%)
COVID-19 complications	
Yes	47 (48.5%)
No	50 (51.5%)
Family member or relative infected	
Yes	91 (93.8%)
No	6 (6.2%)

Coping with diabetes during the pandemic

During the lockdown, 42.3% (n=190) patients were going on walks and doing some physical exercise, and 57.7% (n=259) did not exercise or walk. From the patient responses, we found that only 21.6% (n= 97) participants got the COVID-19 infection, and 78.4% (n=352) did not get infected. Complications from the COVID-19 infection were reported in 12% (n=54) patients, and the majority of respondents reported that they did not have any complications from the COVID-19 virus (n=395; 88%). In addition, relatives who got the infection were 61.5% (n=276), and 38.5% (n=173) did not get infected.

During quarantine, 78.8% (n=354) of participants measured their blood glucose, and only 12% (n=54) did not measure it. About 30.7% (n=138) of the participants thought that monitoring the glucose level was extremely helpful, but only 7.1% (n=32) thought that it wasn't. Nearly half of the participants (n=231; 51.4%) did not measure the HbA1C during quarantine whereas 43.3% (n=154) measured it once or twice, 6.9% (n=31) measured it three times, and 7.3% (n=33) measured it more than three times. Results showed that during quarantine, 68.3% (n=311) participants did not visit their doctor whereas 30.7% (n=138) visited their doctor, and 67.9% (n=305) of the participants were always taking their medication as prescribed. Only 5.1% (n=23) of them never took their medication as prescribed.

Almost all the participants (n=380; 84.6%) did not work outside their home during quarantine and only 15.4% (n=68) were found to be working outside. Less than half of the participants (n=190; 42.3%) reported going on walks during quarantine during the allowed time, while 57.7% (n=258) did not play any sport or go for a walk during the quarantine period. The results showed that 44.1% (n=198) of participants purchased their medication from a community pharmacy, 28.5% (n=128) got their medication by home delivery, 20.9% (n=94) already had a prescription and only 6.5% (n=29) did not need medication during quarantine.

Most of the participants (n=354; 78.7%) did not stop taking their medication and did not have any barrier that prevented them from taking their medication, whereas only 10.2% (n=46) did not know how to get their medications. Around 5.8% (n=26) ran out of their medication and 5.1% (n=23) did not take their medication because of unaffordability.

The present study found a high prevalence of COVID-19 infection among diabetes patients in Saudi Arabia. Almost one-fifth (n=97, 21.6%) of the patients enrolled in the study got the SARS-CoV-2 infection, and 12% of them had COVID-19 complications (Table 2).

**Table 2:** Descriptive analysis and gender differences about diabetic care during the COVID-19 lockdown

Variables	Total		Male		Female			χ^2^	p-value
	N	%	n	%	n		%		
Did you measure your blood glucose during quarantine?									
Yes	354	78%	123	34.7%	231		65.3%	1.546	.462
No	54	12%	21	38.9%	33		61.1%		
Did you get COVID-19 virus infection?									
Yes	97	21.6%	38	39.2%	59		60.8%	.514	.473
No	352	78.4%	124	35.2%	228		64.8%		
If you got COVID-19 infection, did you get any complications?									
Yes	54	12%	18	33.3%	36		66.7%	0.201	.654
No	395	88%	144	36.5%	251		63.5%		
Did any of your family members or relatives get COVID-19?									
Yes	276	61.5%	95	34.4%	181		65.6%	0.856	.355
No	173	38.5%	67	38.7%	106		61.3%		
Did you work outside the home during quarantine?									
Yes	69	15.4%	44	63.8%	25		36.2%	27.101	.000
No	380	84.6%	118	31.1%	262		68.9%		
Were you practicing walking or sports during quarantine?									
Yes	190	42.3%	80	42.1%	110		57.9%	5.185	.023
No	259	57.7%	82	31.7%	177		68.3%		
Did you visit your doctor during quarantine?									
Yes	138	30.7%	52	37.7%	86		62.3%	.221	.638
No	311	69.3%	110	35.4%	201		64.6%		

Participants' attitude towards diabetes

Most of the participants were not worried about the future complications of diabetes, but they agreed that their efforts in taking care of their disease would result in good clinical outcomes (Table 3).

**Table 3:** A descriptive analysis of participants' attitude towards diabetes 7, strongly agree; 6, agree; 5, somewhat agree; 4, neutral; 3, somewhat disagree; 2, disagree; 1, strongly disagree

Variable	N	Mean	Standard deviation
To what extent do your family and friends help you follow your diabetes treatment plan?	449	4.66	2.251
To what extent do you worry about future complications due to your diabetes?	449	4.64	2.205
To what extent do you believe that the benefits of taking care of your diabetes are worth the effort?	449	5.99	1.668

## Discussion

The condition of an intermittent lockdown in order to prevent the spread of COVID-19 infection may have affected the lifestyle, and ultimately, the glycemic control in diabetic patients [16]. A healthy lifestyle, including regular exercise, healthy diet, self-monitoring of blood glucose, and sound sleep, is important to have good immunity and glycemic control that helps to prevent complications related to diabetes mellitus [17]. Diabetic patients are more vulnerable to severe COVID-19 complications with a greater risk [18,19]. Therefore, this study aimed to describe the self-care practices and outcomes in diabetic patients during COVID-19. Our findings indicate that most participants had sufficient knowledge and awareness of their disease. Additionally, most of them were aware of diabetic complications in the case of poor compliance and how the poorly managed diabetes would lead to serious health problems in the future and may reduce their quality of life. Nevertheless, most of our participants had high education levels (high school and above) [20]. A study carried out in Saudi Arabia showed general low to moderate knowledge about diabetes mellitus, its risk factors, and its complications among the Saudi population. As shown, the increasing knowledge and awareness of diabetic patients of their disease in the population will result in better community health outcomes [21]. As COVID-19 affected a large proportion of society and spread rapidly, various measures were implemented at the personal and social levels such as limiting the daily activity and social activity in order to slow down virus transmission. A retrospective study carried out in Saudi Arabia by Khan et al. reported a worse association between clinical outcomes of diabetes and COVID-19 [22]. Diabetes was seen in 11.3% of the cases enrolled in the study, with 20.8% in the critical arm versus 10% in the non-critical arm with a statically significant p-value of 0.005. Our data also revealed that almost a quarter of the sample was only working during quarantine. This gives an impression of how the COVID-19 plus the diabetes affected the working environment. More than half of the sample did not do basic routine exercises, which would impact the disease's progress.

These findings are consistent with other studies showing that the COVID-19 pandemic and lockdown created new obstacles for the psychological health and day-to-day routines of people to resume work during an outbreak [23]. The lockdown and isolation produced drastic aftereffects in terms of social, economic and health conditions of workers of all ages, particularly those with high vulnerabilities and susceptibility to the virus even after returning to work [24,25]. In the present study, the enrolled patients still had several problems in obtaining their medication. There were major changes observed in the healthcare delivery system made by the government for other diseases, including a delivery of medication service and public awareness campaigns reaching out to the press and the public through all possible outlets, especially on social platforms because government was putting a great deal of efforts at all levels against COVID-19 to control its spread [26,27].

In this study, we tried to identify the compliance of diabetes patients to medications during the COVID-19 lockdown in Saudi Arabia. Nonetheless, several limitations were present in this study. First, obtaining the data through face-to-face interviews was difficult due to the fear of the spread of the COVID-19 virus. Although an online self-report survey was necessary to prevent the spread of the virus during the pandemic, it might have also resulted in reporting bias. Second, we cannot generalize the results to all populations because most of the respondents were female and the majority of patients were with type 2 diabetes. But the results, along with the other available literature, can still help in understanding chronic disease patients' self-care practices. Third, this survey was conducted in January 2021 after the second wave of the COVID-19 pandemic; thus, the response was probably dependent on the participant's memory and may not reflect the exact attitude during the COVID-19 lockdown.

## Conclusions

In this study, we found good levels of self-monitoring of blood glucose levels by diabetic patients, whereas patients’ accessibility to seek healthcare services seemed to be interrupted. The study findings could help healthcare providers in providing better diabetes care in the post-pandemic era including the implementation of telehealth and digital interventions to empower self-care behaviors among diabetic patients. For the better prevention and control of diabetes, a comprehensive approach is needed, especially in challenging times such as that of the recent pandemic. Pharmacist-led diabetes care has been altered with the extensive use of virtual and online platforms for service delivery in order to fulfill the needs of patients during the pandemic. The role of pharmacists and other healthcare providers in diabetes care can be further improved by taking a broader view of the facilitators and determinants of the successful implementation of these services in the future studies. Further efforts are needed in the post-pandemic era to empower patients’ self-care behaviors and utilize telehealth models to facilitate timely access to medical care.
